# Low triiodothyronine (T3) levels predict worse outcomes in autoimmune encephalitis—A meta‐analysis of current literature

**DOI:** 10.1002/brb3.3603

**Published:** 2024-06-19

**Authors:** Syeda Tayyaba Rehan, Hassan ul Hussain, Eman Ali, Farea Eqbal, Jawad Ahmed, Mohammed Mahmmoud Fadelallah Eljack, Muhammad Sohaib Asghar

**Affiliations:** ^1^ Department of Medicine Dow University of Health Sciences Karachi Pakistan; ^2^ Faculty of Medicine and Health Sciences University of Bakhtalruda Al‐Dewaym Sudan; ^3^ Department of Medicine Mayo Clinic Rochester Minnesota USA

**Keywords:** antibody, antithyroid, autoimmune, encephalitis, meta‐analysis

## Abstract

**Introduction:**

An unusual association between thyroid dysfunction and autoimmune encephalitis (AE) was noticed when patients presented with low free triiodothyronine (fT3) levels and antithyroid antibodies. We conducted a meta‐analysis to investigate whether thyroid dysfunction, that is, lower fT3 levels are associated with worsening clinical manifestations and prognosis in patients with AE.

**Methods:**

Literature search of five electronic databases was performed till April 5, 2023. Inclusion criteria were as follows: Observational studies reporting patients with all subtypes of AE and assessing thyroid dysfunction categorized as low fT3 and non‐low fT3. Primary endpoints included modified Rankin scale (mRS) at admission, abnormal magnetic resonance imaging, length of stay, seizures, and consciousness declination.

**Results:**

Comprehensive literature search resulted in 5127 studies. After duplicate removal and full‐text screening, six observational studies were included in this analysis. Patients with low fT3 were 2.95 times more likely to experience consciousness declination (*p* = .0003), had higher mRS at admission (*p* < .00001), had 3.14 times increased chances of having a tumor (*p* = .003), were 3.88 times more likely to experience central hypoventilation, and were 2.36 times more likely to have positivity for antithyroid antibodies (*p* = .009) as compared to patients with non‐low fT3.

**Conclusion:**

The findings of our study suggest that low fT3 levels might be related to a more severe disease state, implying the significance of thyroid hormones in AE pathogenesis. This finding is crucial in not only improving the early diagnosis of severe AE but also in the efficient management of the disease.

## Graphical abstract

The findings of our study suggest that low fT3 levels might be related to a more severe disease state, implying the significance of thyroid hormones in Autoimmune Encephalitis pathogenesis. This finding is crucial in not only improving the early diagnosis of severe autoimmune encephalitis but also in the efficient management of the disease.

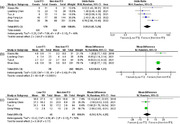

## LIMITATIONS

Several limitations merit consideration in the interpretation of the results of the present study. First, the comparatively moderate sample size may not only limit the statistical power of the paper but may also prevent the results from being extrapolated in the general population. Second, we only evaluated serum thyroid hormones at a single time point. The level of fT3 in critical illness changes over time, which is a dynamic process (Bernal, 2007; Peeters et al., 2005). Future studies evaluating multiple time points are required for validating the predictive role of fT3 in AE.

## 1 INTRODUCTION

Autoimmune encephalitis (AE) refers to a spectrum of disorders in which the host immune system attacks self‐antigens expressed in the central nervous system (CNS), leading to a variety of neurological manifestations, such as seizures, movement disability, speech disorder, cognitive impairment, diminished consciousness, and autonomic dysfunctions (Dalmau & Graus, 2018; Uy et al., 2021). The incidence of AE is comparable to that of infectious encephalitis (13.7 vs. 11.6 per 100,000, respectively) (Silky Pahlajani, 2018). AE incidence has tripled over two decades, from 0.4 in 1995–2005 to 1.2 in 2006–2015, owing mostly to the increasing detection of neural‐specific IgG‐associated encephalitis (Silky Pahlajani, 2018).

AE is further classified into three categories according to antibodies produced against target antigens, such as intracellular antigens, synaptic receptors, and ion channels or other cell‐surface receptors (Graus et al., 2016). The distinction among these three categories is crucial because, although some triggers are identical, their pathogenic mechanisms, clinical profiles, and outcomes differ (Fang et al., 2017). The antibodies against intracellular antigens include anti‐HU type I, anti‐Ma2, and anti‐GAD; antibodies against synaptic receptors include anti‐NMDA, anti‐AMPA, anti‐GABA, anti‐mGluR5, and anti‐D2; and antibodies against ion channels and other cell‐surface proteins include anti‐LGI1, anti‐CASPR2, anti‐MOG, and anti‐aquaporin 4 (Graus et al., 2016). Anti‐NMDA receptor encephalitis is the most prevalent type of AE and has an incidence of 1.5 per million population per year (Dalmau et al., 2007).

An unusual association between thyroid dysfunction and AE was noticed in the past few years when patients of anti‐NMDA encephalitis presented with low free triiodothyronine (fT3) levels and antithyroid antibodies (ATAbs). Antibodies have been found against TSH receptor (TRAb), thyroid peroxidase (TPOAb), and thyroglobulin (TgAb) (Vargas‐Uricoechea et al., 2023). Such patients were repeatedly misdiagnosed with Hashimoto encephalopathy (HE) (Khoodoruth et al., 2020; Mattozzi et al., 2020; Mirabelli‐Badenier et al., 2014; Wu et al., 2023), which is steroid‐responsive encephalitis, which is confirmed by the presence of ATAbs as well as deranged thyroid functions (Schiess & Pardo, 2008). Most patients have elevated thyroid antibodies, which are necessary for diagnosis and include anti‐TPO (thyroid peroxidase) or antithyroglobulin. Out of 105 individuals with HE, 100% had high anti‐TPO antibodies and 48% had raised antithyroglobulin antibodies (Grani et al., 2015; Weetman, 2021). The initial diagnosis strategy is primarily based on neurological examination and conventional diagnostics that are available to most practitioners, as autoantibody test results and responses to therapy are not available at the time of disease onset (Graus et al., 2016).

ATAbs (Lin et al., 2018) and thyroid hormone alterations (Ma et al., 2018) in adult anti‐NMDA encephalitis have been linked to disease severity in recent studies (Ji et al., 2021; Ma et al., 2018; Wang et al., 2022). According to recent studies, fT3 levels were recorded as a significant prognostic marker in other neurological disorders, such as acute ischemic stroke (Song et al., 2022), Alzheimer's disease (AD) (Chiaravalloti et al., 2017), and acute disseminated encephalomyelitis (Wang et al., 2020). Investigations in the clinical practice of treating AE have demonstrated that patients with relatively low fT3 upon admission deteriorated earlier and eventually attained an unsatisfactory outcome (Ji et al., 2021; Lin et al., 2022; Ma et al., 2018; Qiao et al., 2022; Wang et al., 2022). Currently, there are limited data on this subject, and the evidence gathered is rather inconclusive (Lin et al., 2022; Ma et al., 2018; Qiao et al., 2022; Wang et al., 2022). Thus, we conducted a meta‐analysis to investigate whether thyroid dysfunction, that is, lower fT3 levels are associated with the worsening of clinical manifestations and prognosis in patients with AE.

## 2 METHODOLOGY

The present systematic review and meta‐analysis were performed in adherence to the Cochrane collaboration guidelines and reported according to the preferred Reporting Items for Systematic Reviews and Meta‐analysis guidelines (Moher et al., 2009). This paper has been registered on Prospero (ID CRD42023415391).

### 2.1 Data sources and search strategy

Two independent reviewers identified relevant studies through a comprehensive literature search of five electronic databases, including PubMed, Cochrane Library, Google Scholar, Clinicaltrials.gov, and ScienceDirect from inception until April 5, 2023. Predefined MesH terms were utilized by applying Boolean operators (“AND” and “OR”). The search was devoid of any time constraints, and articles published in languages other than English were excluded. The search strategy consisted of a combination of the following keywords: “encephalitis” OR “autoimmune encephalitis” OR “anti‐*N*‐methyl‐d‐aspartate receptor encephalitis” OR “anti‐NMDA receptor encephalitis” AND “thyroid dysfunction” OR “fT3 levels” OR “thyroid antibodies.” Bibliographies of the included original articles and pertinent review articles were also reviewed.

### 2.2 Study selection and eligibility criteria

All search results were imported into Mendeley Desktop 1.19.8 (Mendeley Ltd.), followed by the removal of duplicates. Abstracts and full texts were reviewed by two independent reviewers (STR, HUH). In case of any disagreement, a third reviewer was consulted.

The pre‐established inclusion criteria were as follows: (1) observational studies (prospective and retrospective) reporting patients with overall AE and other antibody types, that is, anti‐NMDA, anti‐LGI, anti‐GABA type B, anti‐CASPR2, anti‐AMPA, anti‐MOG, anti‐GAD 65, and anti‐amphiphysin; (2) studies assessing thyroid dysfunction categorized as low fT3 and non‐low fT3. Low fT3 is defined as a group of AE patients having fT3 levels lesser than 3.39 pmol/L (2.43–7.50 pmol/L) or patients with the euthyroid sick syndrome. Although non‐low fT3 group patients were the ones with either high or normal fT3 levels, (3) studies that reported outcomes of interest. The primary endpoints were modified Rankin scale (mRS) at admission, abnormal magnetic resonance imaging (MRI), length of stay (LOS), seizures, and consciousness decline. The secondary endpoints, such as tumor presence, speech disorder, movement disorder, autonomic dysfunction, psychiatric behavior, cognitive dysfunction, central hypoventilation, and thyroid antibody positivity.

Case reports, letters, reviews, commentaries, studies that did not correlate thyroid dysfunction with clinical outcomes, and patients with other nervous system disorders (such as viral encephalitis) were excluded.

### 2.3 Data extraction

Information about study details, type of AE, patient demographics, outcome measures, total population, and control in the study were extracted by two independent groups of authors. The number of events for the outcome and the total number of patients were retrieved for categorical outcomes, and for continuous outcomes, the sample size and mean or median with standard deviation were extracted.

### 2.4 Quality assessment

The two independent reviewers (EA and FE) assessed the risk of bias and quality of the included studies using the Newcastle–Ottawa scale (Wells et al., 2018). Studies with a total score of 8 or 9 had a predicted low risk of bias, whereas studies with a total score of 6 or 7 had a predicted medium risk of bias. A higher score denotes an improved methodological approach.

### 2.5 Statistical analysis

The outcomes were pooled with the Mantel–Haenszel random‐effects model using Review Manager (Version 5.3, The Cochrane Collaboration). When applicable, continuous data were presented as mean difference and 95% confidence interval (CI). The odds ratios (OR) with 95% CI were used to present dichotomous data. A *p*‐value <.05 was considered statistically significant. To examine heterogeneity, the Cochrane *Q* statistic and the inconsistency factor (*I* ^2^) were employed (Higgins et al., 2019). Significant heterogeneity was defined as an *I* ^2^ value >75% with *I* ^2^ values of 25%–50% as low, 50%–75% as moderate, and >75% as a high degree of heterogeneity. Leave‐one‐out sensitivity analyses were done to find out if any study was driving the results or if there was high heterogeneity.

## 3 RESULTS

### 3.1 Literature search

**FIGURE 1:**
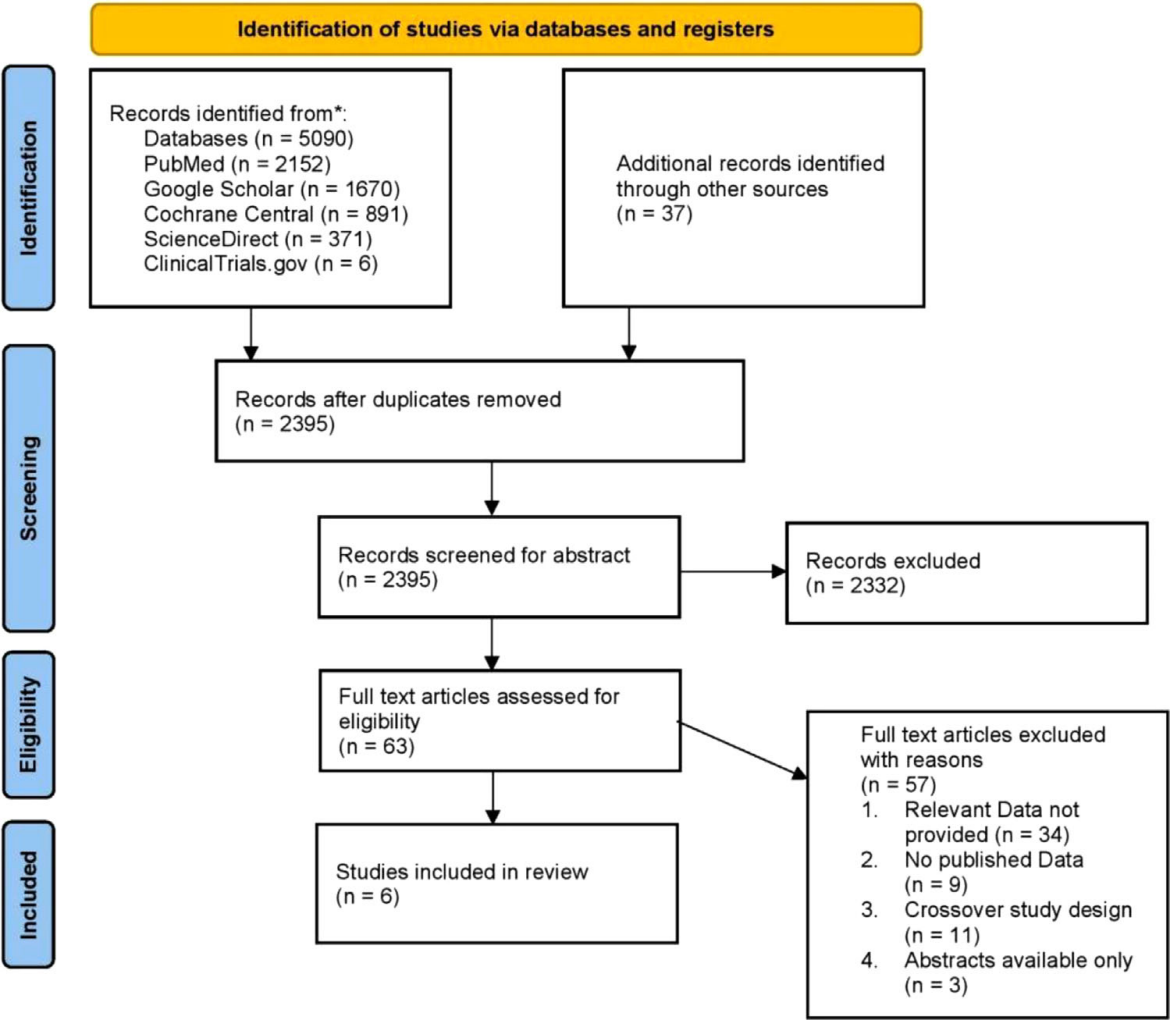
PRISMA flowchart of literature search.

An initial search provided 5127 studies, and after the removal of duplicates, 2395 records remained. After a full‐text review of 63 articles that were assessed for their eligibility, 6 observational studies were finalized for this meta‐analysis. A complete literature search has been highlighted in the PRISMA flowchart in Figure 1.

### 3.2 Study characteristics

Study characteristics and patients’ demographics have been mentioned in detail in Tables 1 and 2, respectively. Out of six observational studies (Chen et al., 2021; Ji et al., 2021; Lin et al., 2022; Ma et al., 2018; Qiao et al., 2022; Wang et al., 2022), five of them (Chen et al., 2021; Lin et al., 2022; Ma et al., 2018; Qiao et al., 2022; Wang et al., 2022) had a retrospective design, whereas one was prospective (Ji et al., 2021) in nature. A total of 683 patients were enrolled in these 6 studies, out of which 191 patients belonged to the low fT3 group, whereas 492 patients belonged to the non‐low fT3 group. The mean age of the patients ranged from 6.2 to 34.5 years, with an average of 29.9 years. The percentage of males ranged from 23.1 to 57.8, with a mean of 45.8% of the total population.

**TABLE 1:** Study characteristics.

References	Study type	Region/Hospital	Duration of study	Total study population	Control
Ma et al. (2018)	Retrospective observational study	Shandong Provincial Hospital affiliated to Shandong University	January 2014 to January 2018	268	A total of 225 healthy subjects in health examination center
Chen et al. (2021)	Retrospective observational study	Guangzhou Women and Children's Medical Center	August 2016 to 2019	51	–
Ji et al. (2021)	Prospective observational study	Zhengzhou University First Affiliated Hospital	1 January 2016 and 31 December 2019	96	–
Lin et al. (2022)	Retrospective observational study	Neurology center, West China Hospital	June 2012 to September 2020	450	A total of 229 subjects who visited our hospital for a health examination
Wang et al. (2022)	Retrospective observational study	(Xinqiao Hospital, Army Medical University, Chongqing, China)	September 2014 to June 2021	103	–
Qiao et al. (2022)	Retrospective observational study	Qilu Hospital, Cheeloo College of Medicine, Shandong University, China	January 2016 to December 2020	237	–

**TABLE 2:** Population characteristics.

References	Type of autoimmune encephalitis	Total population	Population with low fT3	Population with non‐low fT3	Age (mean ± SD)	Sex, male/female (*N*)
Ma et al. (2018)	Anti‐NMDA	42	11	31	29.65 ± 12.18	11/32
Chen et al. (2021)	Anti‐NMDA	51	23	28	–	23/28
Ji et al. (2021)	Anti‐NMDA	96	32	64	–	53/43
Lin et al. (2022)	A total of 156 patients with NMDAR antibodies, 41 patients with LGI1 and/or CASPR2 antibodies, and 24 patients with GABABR antibodies were detected	221	77	144	34.50 ± 15.58	93/128
Wang et al. (2022)	Anti‐NMDA	36	12	24	38 ± 16	12/24
Qiao et al. (2022)	107 patients with anti‐NMDAR, 86 with anti‐LGI1, 22 with anti‐GABAB, and 22 with miscellaneous subtypes of AE; these being anti‐CASPR2 (9 cases), anti‐AMPA, anti‐MOG, anti‐GAD 65, and anti‐amphiphysin	237	36	201	–	36/201

Abbreviations: AE, autoimmune encephalitis; fT3, free triiodothyronine.

### 3.3 Quality assessment and publication bias

Based on the quality assessment scale, five observational studies were rated as “Good,” and one study was rated as “Fair” quality (Table S1). Wang et al. (2022) was rated as “Fair” quality as this study was retrospective and failed to perform univariate/multivariate regression analysis to account for sex, age, gender, and other confounders in the study.

### 3.4 Outcome analysis

**FIGURE 2:**
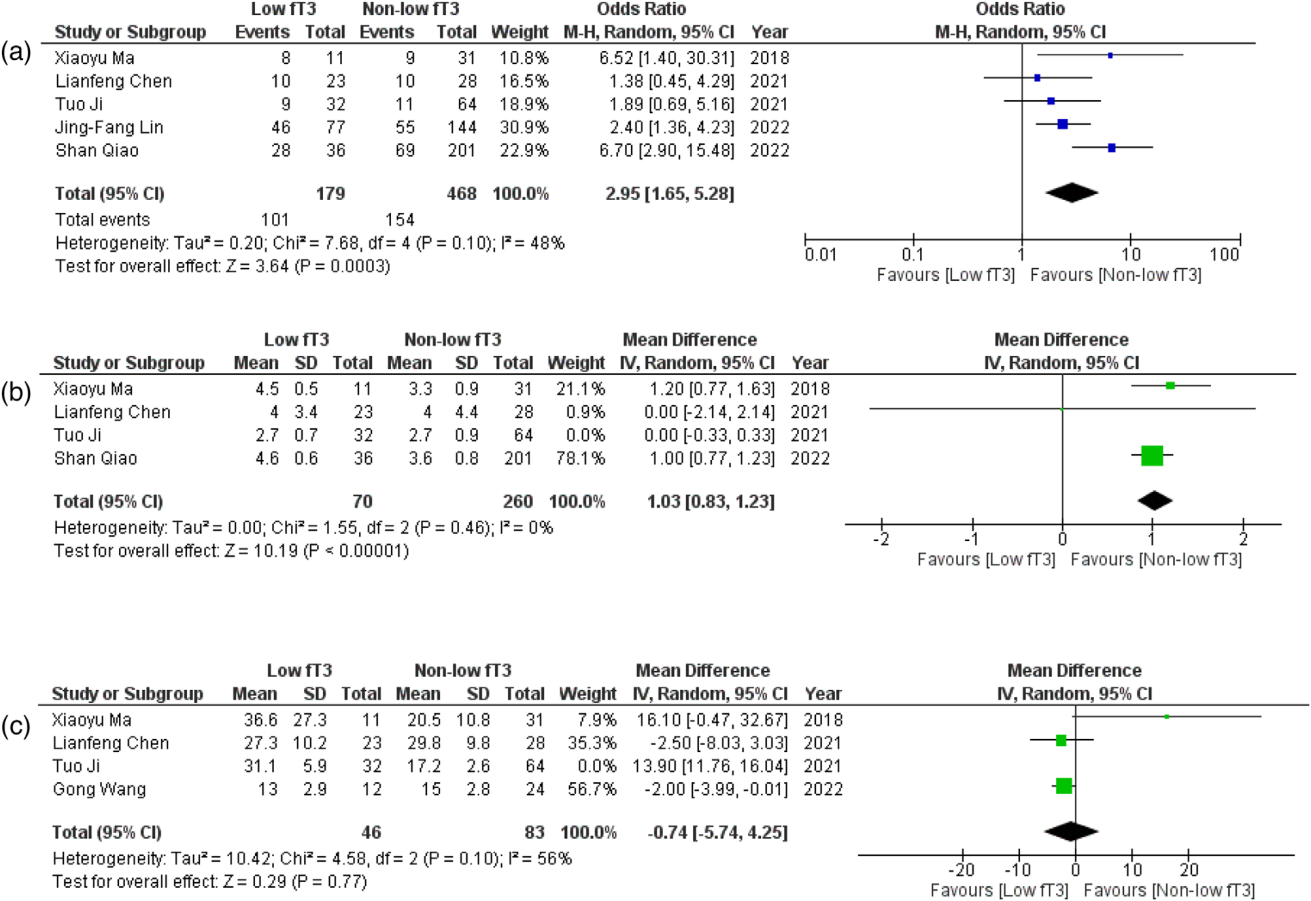
(a) Forest plot depicting odds ratios for consciousness declination; (b) forest plot depicting mean differences for modified Rankin scale (mRS) at admission after sensitivity analysis; (c) forest plot depicting mean differences for the length of stay at the hospital after sensitivity analysis.

**FIGURE 3:**
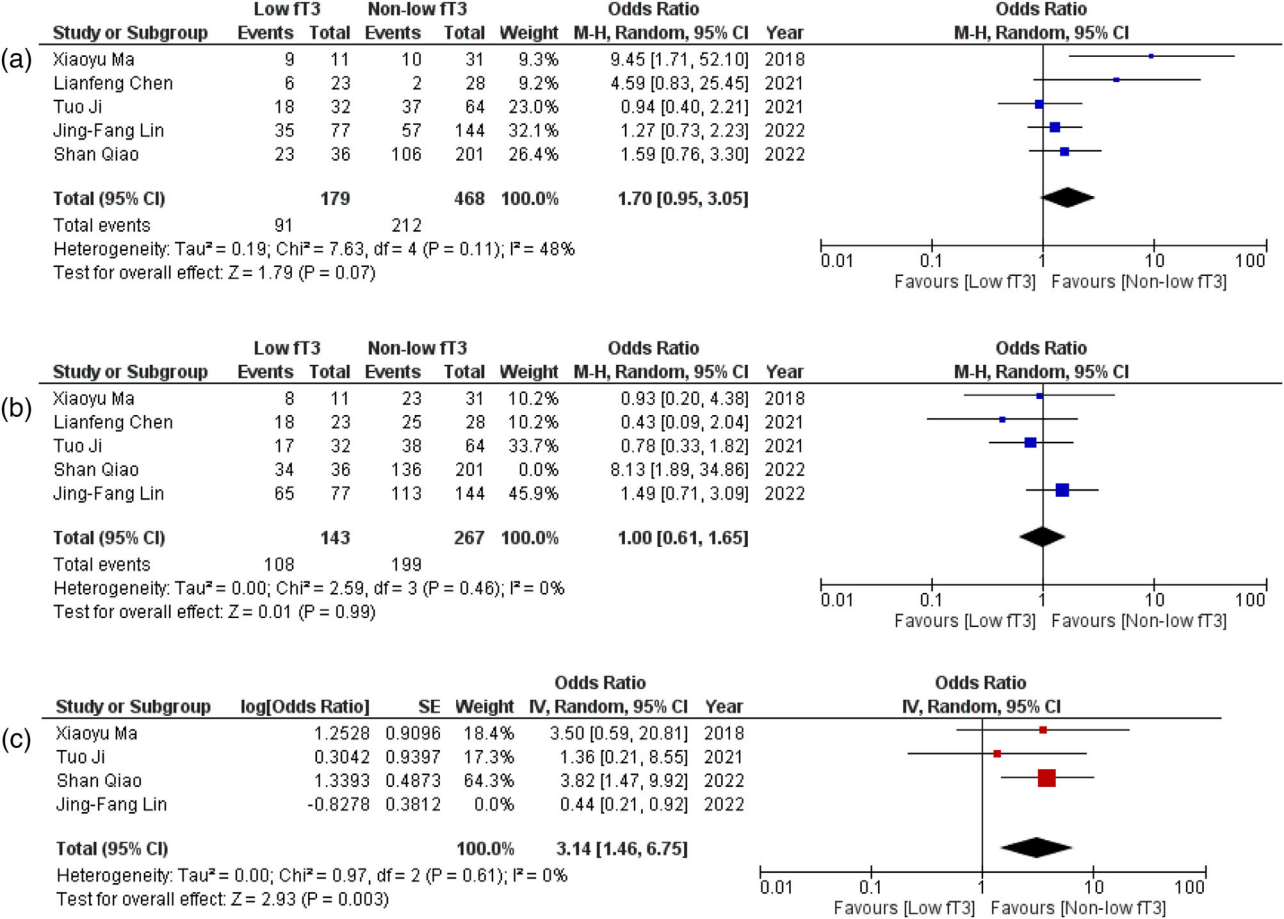
(a) Forest plot depicting odds ratios for magnetic resonance imaging (MRI) abnormality; (b) forest plot depicting odds ratios for seizures after sensitivity analysis; (c) forest plot depicting odds ratios for tumor presence after sensitivity analysis.

**FIGURE 4:**
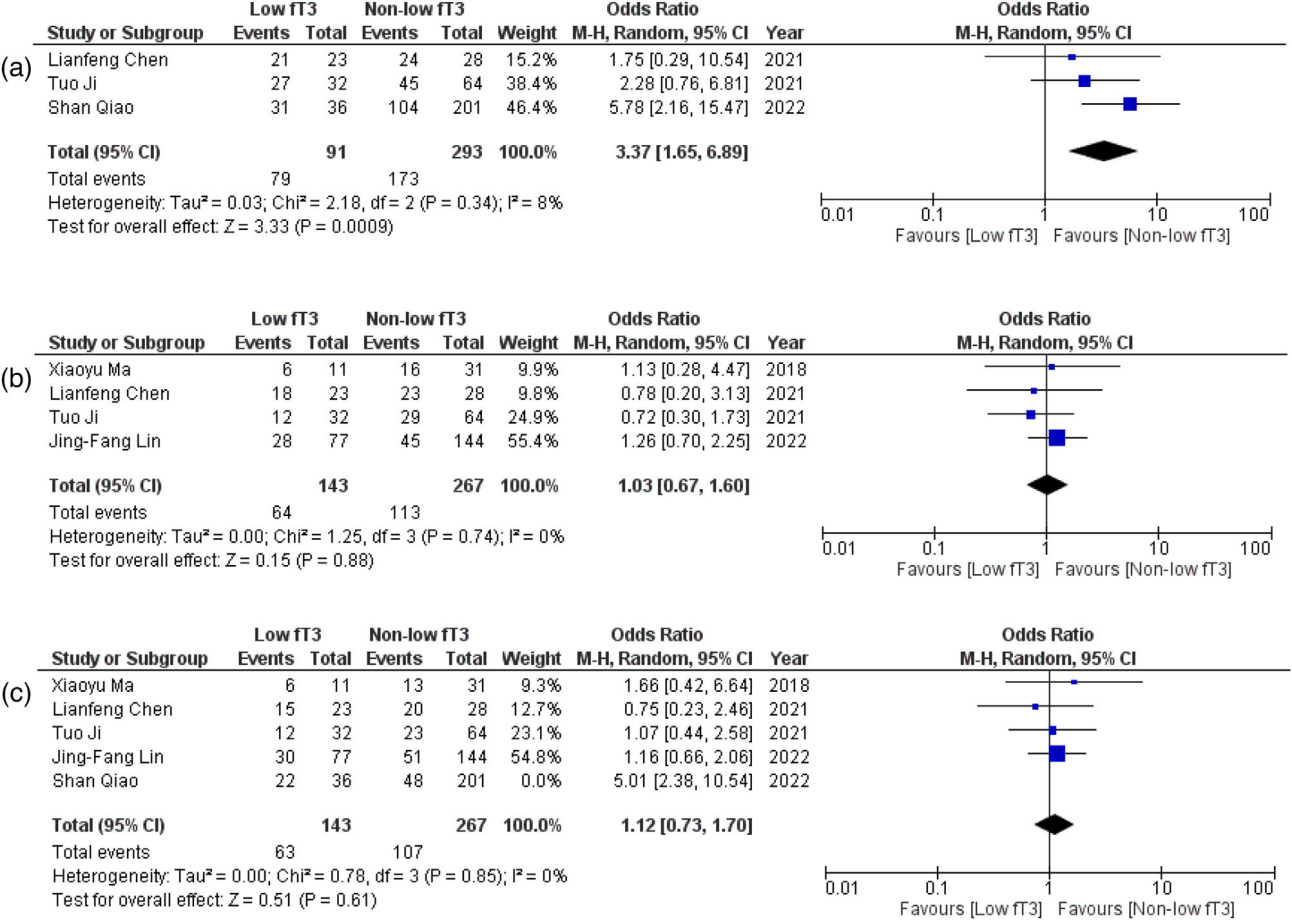
(a) Forest plot depicting odds ratios for psychiatric behavior; (b) forest plot depicting odds ratios for speech disorder; (c) forest plot depicting odds ratios for movement disorders after sensitivity analysis.

**FIGURE 5:**
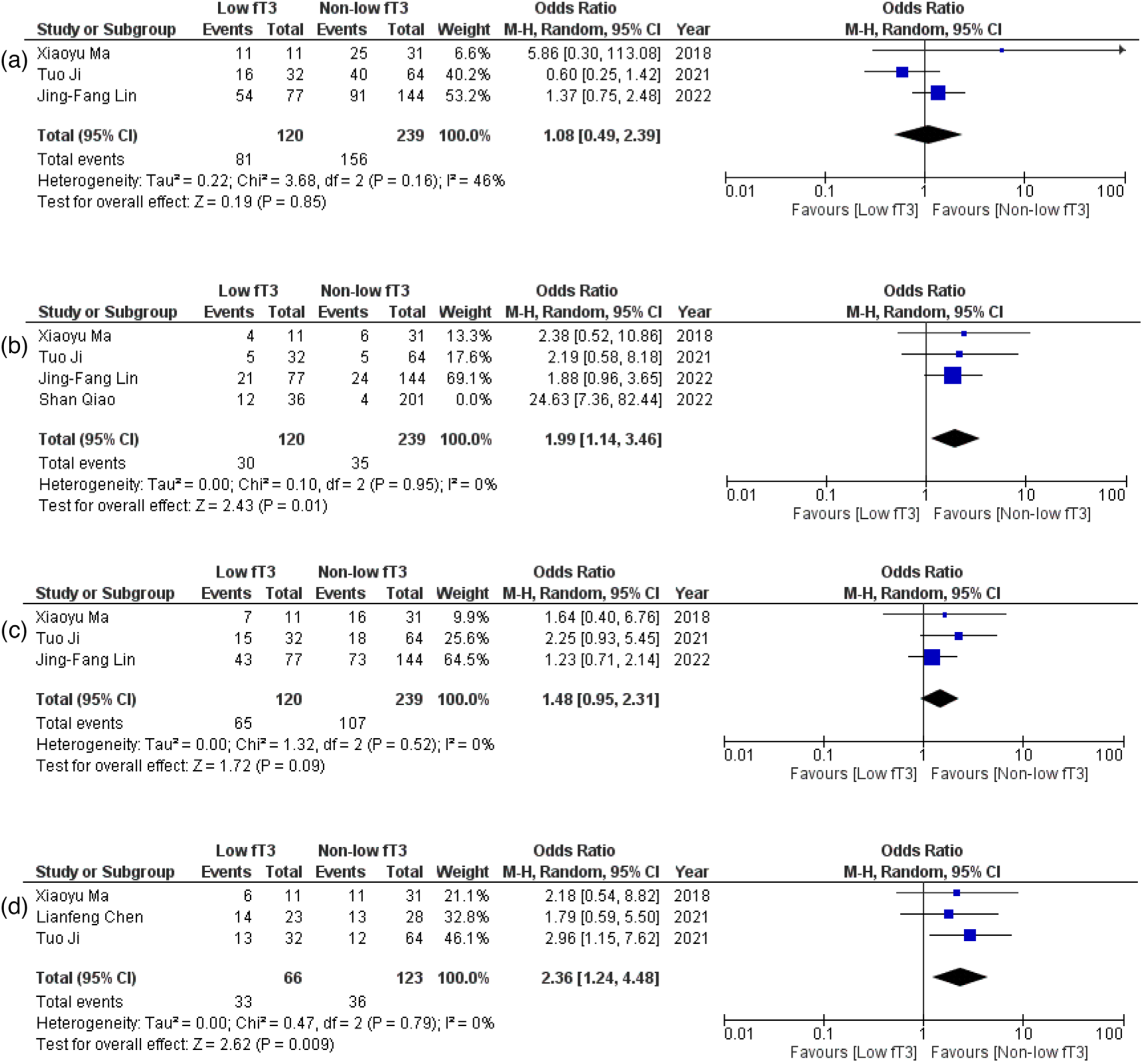
(a) Forest plot depicting odds ratios for cognitive dysfunction; (b) forest plot depicting odds ratios for central hypoventilation after sensitivity analysis; (c) forest plot depicting odds ratios for autonomic dysfunction; (d) forest plot depicting odds ratios for antithyroid antibody positivity.

All six observational studies reported the correlation of fT3 levels with several outcomes in patients of AE. Detailed forest plots with effect sizes of primary and secondary outcomes are given in Figures 2, 3, 4, 5.

### 3.5 Primary outcomes

#### 3.5.1 Consciousness declination

Five studies (Chen et al., 2021; Ji et al., 2021; Ma et al., 2018; Qiao et al., 2022; Song et al., 2022) provided data for the relation between fT3 levels and consciousness declination. Pooled analysis revealed that patients with low fT3 were significantly more likely to experience consciousness declination than the patient with non‐low fT3 (OR = 2.95; 95% CI = 1.65–5.28; *p* = .0003, *I* ^2^ = 48%) (Figure 2).

#### 3.5.2 mRS at admission

Out of six studies, four (Chen et al., 2021; Ji et al., 2021; Ma et al., 2018; Qiao et al., 2022) provided data on mRS at admission. The pooled analysis showed a significant relationship between fT3 levels and mRS at admission (WMD = .67; 95% CI = .01–1.34; *p* = .05, *I* ^2^ = 90%) (Figure S1) (removal of Ji et al. (2021) via sensitivity analysis) (Figure 2) (WMD = 1.03; 95% CI = .83–1.23; *p* < .00001, *I* ^2 ^= 0%).

#### 3.5.3 Length of stay at hospital

Four (Chen et al., 2021; Ji et al., 2021; Ma et al., 2018; Wang et al., 2022) studies reported data on the LOS at hospital in relation with fT3 levels. No significant difference was evidenced between days spent at hospital when patients of low fT3 were compared with non‐low fT3 (WMD = 5.45; 95% CI = −5.47 to 16.37; *p* = .33, *I* ^2^ = 98%) (Figure S2). Removal of Ji et al. (2021) reduced heterogeneity (WMD = −0.74; 95% CI = −5.74 to 4.25; *p* = .77, *I* ^2 ^= 56%) (Figure 2).

#### 3.5.4 Abnormal MRI

Pooled data from five (Chen et al., 2021; Ji et al., 2021; Lin et al., 2022; Ma et al., 2018; Qiao et al., 2022) studies showed no significant difference in the abnormality of MRI when low and non‐low fT3 levels patients were compared (OR = 1.70; 95% CI = .95–3.05; *p* = .07, *I* ^2^ = 48%) (Figure 3).

#### 3.5.5 Seizures

Five (Chen et al., 2021; Ji et al., 2021; Lin et al., 2022; Ma et al., 2018; Qiao et al., 2022) studies reported data on the presence of seizures. No statistically significant association was reported between fT3 levels and the occurrence of seizures (OR = 1.28, 95% CI = .57–2.91, *p* = .55, *I* ^2^ = 61%) (Figure S3). However, sensitivity analysis was performed by removing a single study (Song et al., 2022), which did not change the results much (OR = 1.00; 95% CI = .61–1.65; *p* = .99, *I* ^2^ = 0%), but a substantial decrease was observed in heterogeneity (Figure 3).

### 3.6 Secondary outcomes

#### 3.6.1 Presence of tumor

Out of six included studies, four (Ji et al., 2021; Lin et al., 2022; Ma et al., 2018; Qiao et al., 2022) provided data on the presence of tumors in association with fT3 levels. No significant association was noted between fT3 levels and the presence of tumor in those patients (OR = 1.57; 95% CI = .43–5.81; *p* = .50, *I* ^2^ = 79%) (Figure S4). There was a significant shift in the values after we performed sensitivity analysis by removing one study (Lin et al., 2022). The three studies now revealed that patients with low fT3 were 3.14 times more likely to have the presence of tumor (OR = 3.14; 95% CI = 1.46–6.75; *p* = .003, *I* ^2^ = 0%) and revealed no heterogeneity of the included studies (Figure 3).

#### 3.6.2 Psychiatric behavior

The pooled analysis of data from three studies (Chen et al., 2021; Ji et al., 2021; Qiao et al., 2022) showed an important correlation between fT3 levels and psychiatric behavior. It showed that patients with low fT3 levels were 3.37 times more likely to exhibit psychiatric behavior (OR = 3.77; 95% CI = 1.65–6.89; *p* = .0009, *I* ^2^ = 8%) (Figure 4).

#### 3.6.3 Speech disorders

Adequate data on speech disorders was reported in four (Chen et al., 2021; Ji et al., 2021; Lin et al., 2022; Ma et al., 2018) studies. No significant association was observed between fT3 levels and the presence of speech disorders (OR = 1.03; 95% CI = .67–1.60; *p* = .88, *I* ^2^ = 0%) (Figure 4).

#### 3.6.4 Movement disorders

Sufficient data were provided by five studies (Chen et al., 2021; Ji et al., 2021; Lin et al., 2022; Ma et al., 2018; Qiao et al., 2022) on movement disorders in patients with AE. No significant correlation was evidenced between fT3 levels and patients exhibiting movement disorders (OR = 1.56; 95% CI = .77–3.16; *p* = .21, *I* ^2^ = 68%) (Figure S5). We then performed sensitivity analysis by eliminating one study (20), which did not change the results; however, heterogeneity was considerably decreased (OR = 1.12; 95% CI = .73–1.70; *p* = 0.61, *I* ^2^ = 0%) (Figure 4).

#### 3.6.5 Cognitive dysfunction

No direct significant difference was observed in patients with low fT3 and non‐low fT3 levels in terms of cognitive dysfunction (OR = 1.08; 95% CI = .49–2.39; *p* = .85, *I* ^2^ = 46%) (Figure 5).

#### 3.6.6 Central hypoventilation

Four studies (Ji et al., 2021; Lin et al., 2022; Ma et al., 2018; Qiao et al., 2022) reported data on this outcome. A statistically significant correlation was noted, where patients with low fT3 levels were 3.88 times more likely to experience central hypoventilation as opposed to the ones with non‐low fT3 levels (OR = 3.88; 95% CI = 1.15–13.09; *p* = .03, *I* ^2^ = 78%) (Figure S6). After performing sensitivity analysis and removing one study (Song et al., 2022), our findings turned out to be further significant (OR = 1.99; 95% CI = 1.14–3.46; *p* = .01, *I* ^2^ = 0%) with greatly reduced heterogeneity (Figure 5).

#### 3.6.7 Autonomic dysfunction

Three (Ji et al., 2021; Lin et al., 2022; Ma et al., 2018) studies reported data for this outcome. No significant difference was obtained when patients with low fT3 and non‐low fT3 were compared for experiencing autonomic dysfunction (OR = 1.48; 95% CI = .95–2.31; *p* = .09, *I* ^2^ = 0%) (Figure 5).

#### 3.6.8 Antithyroid antibody status

Out of all, three studies (Ji et al., 2021; Lin et al., 2022; Ma et al., 2018) provided the data that levels of fT3 were related to the status of antithyroid antibodies. A significant correlation was noted where patients with low fT3 were 2.36 times more likely to have positivity for antithyroid antibodies as opposed to patients with non‐low fT3 (OR = 2.36; 95% CI = 1.24–4.48; *p* = .009, *I* ^2^ = 0%) (Figure 5).

## 4 DISCUSSION

In this comprehensive meta‐analysis, we evaluated the association between decreased fT3 levels and the presence of severe adverse clinical outcomes in patients with AE. Data from 6 observational studies consisting of a total of 683 patients were pooled into this meta‐analysis. The patients were grouped according to fT3 levels; out of 683 patients, 191 patients reported low fT3 levels, whereas 492 patients belonged to the non‐low fT3 group. In summary, the results of our study illustrated a notably greater likelihood of worse prognostic outcomes, such as consciousness declination, high mRS at admission, psychiatric behavior, and central hypoventilation, as compared to the patients in the non‐low fT3 levels group. It was also demonstrated that patients with low fT3 levels were 2.36 times more likely to be ATAb positive in contrast to patients with non‐low fT3 levels.

Accumulating evidence suggests that lower serum T3 concentrations may be associated with greater severity, a more complicated clinical course, higher mortality rates, and an elevated risk for poor functional outcomes at discharge and in the long term in patients with acute cerebrovascular events, respiratory failure, and brain tumors (Bunevicius et al., 2015). The pivotal role of fT3 levels in evaluating the prognosis and severity of a wide range of diseases has been assessed in numerous meta‐analyses (Chen et al., 2022; Dhital et al., 2017; Dolatshahi et al., 2023; Llamas et al., 2021; Xu et al., 2016) in the past, but there are only a few studies that have previously focused on the relationship between thyroid dysfunction and the functional outcome of AE. Importantly, our data indicated that low fT3 levels upon admission were associated with relatively poor short‐term functional outcomes in AE patients, which was consistent with prior reports and meta‐analyses related to the short‐term prognostic value of low fT3 in some diseases, including encephalitis (Feng et al., 2018), severe brain injury (Olivecrona et al., 2013), and COVID‐19 disease (Chen et al., 2020; Lui et al., 2021; Prakash, 1983). However, it is worth noting that other thyroid hormones, including T3, T4, fT4, and TSH, also play a crucial role in the development and maturation of the mammalian CNS (Valencia‐Sanchez et al., 2021). The condition known as hypothyroidism arises when the thyroid gland is unable to generate enough thyroid hormone. The most frequent cause of hypothyroidism is Hashimoto thyroiditis, a disorder in which autoantibodies target thyroid follicular cells, resulting in a reduction in the production of thyroid hormone (Pirahanchi et al., 2023). Additional common causes of hypothyroidism include pituitary tumors, radiation therapy, thyroid surgery, overuse of antithyroid drugs, congenital hypothyroidism, and iodine shortage. Hypothyroidism symptoms include low basal metabolic rate, weight gain in spite of decreased appetite, sensitivity to cold, low cardiac output, hypoventilation, mental slowness and lethargy, drooping eyelids, myxedema, growth retardation, mental retardation in patients who are pregnant, and goiter (Pirahanchi et al., 2023). TSH levels rise as a result of a lack of negative feedback inhibition in primary hypothyroidism, as well as in Hashimoto thyroiditis (Pirahanchi et al., 2023).

Such consistency in the findings of our study with previous meta‐analyses not only enhances the authenticity of our analysis but also warrants the effectiveness of fT3 levels in evaluating AE severity on the basis of a variety of primary and secondary outcomes, not evaluated in the past. Even though previous meta‐analyses have assessed the prognostic role of low fT levels in assessing the severity of CNS diseases such as AD (Dolatshahi et al., 2023), this is the first meta‐analysis to evaluate the prognostic role of low fT3 levels in assessing AE severity. A retrospective analysis performed on the data of neurocritical patients with low T3 syndrome elucidated that low fT3 levels are a strong predictor of mortality and poor prognosis in critical care patients (Chen et al., 2020). Similarly, another study demonstrated consistent association in terms of low T3 levels in paraplegic and quadriplegic patients, further enhancing the credibility of our hypothesis (Prakash, 1983).

Moreover, the prognostic role of fT3 levels is not only limited to neurologic disorders and COVID‐19 illnesses but also extends to cardiovascular diseases. The results of a meta‐analysis revealed consistently higher cardiovascular mortality in the presence of thyroid hormone level derangements in patients undergoing long‐term dialysis (Xu et al., 2016). Another similar meta‐analysis demonstrated that low initial T3/fT3 correlates with worse outcomes in acute ischemic stroke among clinically euthyroid patients (Dhital et al., 2017).

The mechanism of how the fT3 influences the evolution and outcome of AE remains elusive. A variety of evidence supports the speculation that autoimmune diseases may challenge the bidirectional communication among the central nervous, endocrine, and immune systems (Jara et al., 2013). Such disturbance in homeostatic–molecular balance participates in autoimmunity and in the pathogenesis of autoimmune diseases (Di Comite et al., 2007).

It has been postulated that fT3 has a critical role in the proliferation and differentiation of neuronal progenitors during brain development and the regulation of immune system function (Bernal, 2007; Desouza et al., 2005). Therefore, we speculate that patients with low fT3 might experience decreased neuroprotection and increased secondary brain damage after encephalitis, leading to higher disease severity during hospitalization and poorer outcomes. Another potential explanation for the correlation between low fT3 levels and worse disease prognosis could be that systemic inflammation can reduce deiodinase activity, thereby converting total thyroxine to total triiodothyronine, leading to low fT3 levels (Lui et al., 2021). Moreover, a statistically significant correlation between low fT3 levels and consciousness declination could be explained by the postulation that thyroid hormones participate in the secretion of neurotrophic factors (Kramer et al., 2009). However, it is crucial to provide comprehensive evidence to further authenticate these potential explanations for the role of low fT3 levels in AE outcome severity.

The statistically significant correlation between low fT3 levels and ATAb positivity revealed in our study implies the potential role of thyroid antibodies in AE pathophysiology (Lin et al., 2018). This could be explained by the immunomodulatory effects of ATAbs, which form immune complexes with myelin basic protein that lead to cerebral injuries (Sakuma et al., 1999). It should be acknowledged that no significant difference was observed in the length of hospital stay, abnormal MRI, and seizures between patients with low fT3 and non‐low fT3. This could be attributed to the presence of heterogeneity among the included studies that assessed length of hospital stay (*I* ^2^ = 98%), abnormal MRI (*I* ^2^ = 48%), and seizures (*I* ^2^ = 61%). However, the results remained the same after performing a leave‐one‐out sensitivity analysis, undermining the authenticity of our study.

The frequency and distribution of thyroid autoantibodies, as well as the prevalence of hyperthyroidism and hypothyroidism, have been determined in various, primarily Caucasian communities through cross‐sectional investigations conducted in Europe, the USA, and Japan (Laurberg et al., 2001; Vanderpump et al., 2005). Studies from Europe have shown the impact of dietary iodine intake on the epidemiology of thyroid dysfunction, whereas data from screening large US population samples have shown differences in the frequency of thyroid dysfunction and serum thyroid antibody concentrations in different ethnic groups (Canaris et al., 2000; Hollowell et al., 2002). As most of the studies included Chinese individuals, our results could be interpreted and applicable for Asian races only, and cross‐cultural data would be required to broadly understand the presence of thyroid diseases and thyroid antibodies.

The incidence of AE is on the rise, necessitating timely diagnosis and management based on the prognosis of the clinical manifestations. Therefore, it has become essential to shift the focus of research studies toward evaluating the prognostic markers for AE, clinical manifestations, and functional outcomes. The present study is the first meta‐analysis to assess the diagnostic value of low fT3 levels in association with AE progression and a wide range of outcomes.

## 5 CONCLUSION

In conclusion, our meta‐analysis demonstrates that fT3 levels are implicated in the evolution and progression of AE. The findings of our study suggest that low fT3 levels might be related to a more severe disease state, implying the significance of thyroid hormones in AE pathogenesis. This finding is crucial in not only improving the early diagnosis of severe AE but also in the efficient management of the disease. However, the underlying mechanism explaining the role of fT3 levels in critical AE patients remains unexplored. Therefore, particular attention should be paid to AE patients reporting with low fT3 levels, which might aid clinical prediction and guide clinical decision‐making. Further large‐scale cohort studies are needed to confirm the prognostic value of fT3 in evaluating AE severity.

## AUTHOR CONTRIBUTIONS

**Syeda Tayyaba Rehan**: Conceptualization; investigation; methodology; project administration; writing—review and editing. **Hassan ul Hussain**: Conceptualization; data curation; visualization; writing—original draft. **Eman Ali**: Conceptualization; data curation; formal analysis; validation; writing—original draft. **Farea Eqbal**: Data curation; resources; software; validation. **Jawad Ahmed**: Data curation; formal analysis; investigation; writing—original draft. **Mohammed Mahmmoud Fadelallah Eljack**: Funding acquisition; methodology; supervision; writing—review and editing. **Muhammad Sohaib Asghar**: Project administration; resources; software; writing—review and editing.

## FUNDING INFORMATION

None.

## CONFLICT OF INTEREST STATEMENT

The authors declare no conflicts of interest.

### PEER REVIEW

The peer review history for this paper is available at https://publons.com/publon/10.1002/brb3.3603.

## Supporting information

Table S1 Search strategy used in each database searched.
Figure S1 Forest plot depicting mean differences for mRS at admission before sensitivity analysis.
Figure S2 Forest plot depicting mean differences for length of stay at hospital before sensitivity analysis.
Figure S3 Forest plot depicting odds ratios for seizures before sensitivity analysis.
Figure S4 Forest plot depicting odds ratios for tumor presence before sensitivity analysis.
Figure S5 Forest plot depicting odds ratios for movement disorders before sensitivity analysis.
Figure S6 Forest plot depicting odds ratios for central hypoventilation before sensitivity analysis.

## Data Availability

No new datasets were generated during the conduct of this systematic review; all relevant literature has been included either within the body of the manuscript, supplementary materials, or references.
