# Supplementary material for: Low triiodothyronine (T3) levels predict worse outcomes in autoimmune encephalitis—A meta‐analysis of current literature
Source: Brain Behav. 2024 Jun 19;14(6):e3603. doi: 10.1002/brb3.3603 (PMC11186844; doi:10.1002/brb3.3603)
Supplement: Supplementary file 1 — Table S1 Search strategy used in each database searched. Figure S1 Forest plot depicting mean differences for mRS at admission before sensitivity analysis. Figure S2 Forest plot depicting mean differences for length of stay at hospital before sensitivity analysis. Figure S3 Forest plot depicting odds ratios for seizures before sensitivity analysis. Figure S4 Forest plot depicting odds ratios for tumor presence before sensitivity analysis. Figure S5 Forest plot depicting odds ratios for movement disorders before sensitivity analysis. Figure S6 Forest plot depicting odds ratios for central hypoventilation before sensitivity analysis. [file BRB3-14-e3603-s001.docx]

**Supplementary Table 1: Search strategy used in each database searched.**

| **Database** | **Search Strategy** | **Articles retrieved** |
| --- | --- | --- |
| Pubmed | ((("thyroid gland"[MeSH Terms] OR ("thyroid"[All Fields] AND "gland"[All Fields]) OR "thyroid gland"[All Fields] OR "thyroid"[All Fields] OR "thyroid usp"[MeSH Terms] OR ("thyroid"[All Fields] AND "usp"[All Fields]) OR "thyroid usp"[All Fields] OR "thyroids"[All Fields] OR "thyroid s"[All Fields] OR "thyroidal"[All Fields] OR "thyroideal"[All Fields] OR "thyroidism"[All Fields] OR "thyroiditis"[MeSH Terms] OR "thyroiditis"[All Fields] OR "thyroiditides"[All Fields]) AND ("functional"[All Fields] OR "functional s"[All Fields] OR "functionalities"[All Fields] OR "functionality"[All Fields] OR "functionalization"[All Fields] OR "functionalizations"[All Fields] OR "functionalize"[All Fields] OR "functionalized"[All Fields] OR "functionalizes"[All Fields] OR "functionalizing"[All Fields] OR "functionally"[All Fields] OR "functionals"[All Fields] OR "functioned"[All Fields] OR "functioning"[All Fields] OR "functionings"[All Fields] OR "functions"[All Fields] OR "physiology"[MeSH Subheading] OR "physiology"[All Fields] OR "function"[All Fields] OR "physiology"[MeSH Terms])) OR (("antithyroid"[All Fields] OR "antithyroidal"[All Fields] OR "antithyroids"[All Fields]) AND ("antibodie"[All Fields] OR "antibodies"[MeSH Terms] OR "antibodies"[All Fields] OR "antibody s"[All Fields] OR "antibodys"[All Fields] OR "immunoglobulins"[MeSH Terms] OR "immunoglobulins"[All Fields] OR "antibody"[All Fields])) OR (("thyroid gland"[MeSH Terms] OR ("thyroid"[All Fields] AND "gland"[All Fields]) OR "thyroid gland"[All Fields] OR "thyroid"[All Fields] OR "thyroid usp"[MeSH Terms] OR ("thyroid"[All Fields] AND "usp"[All Fields]) OR "thyroid usp"[All Fields] OR "thyroids"[All Fields] OR "thyroid s"[All Fields] OR "thyroidal"[All Fields] OR "thyroideal"[All Fields] OR "thyroidism"[All Fields] OR "thyroiditis"[MeSH Terms] OR "thyroiditis"[All Fields] OR "thyroiditides"[All Fields]) AND ("autoimmune diseases"[MeSH Terms] OR ("autoimmune"[All Fields] AND "diseases"[All Fields]) OR "autoimmune diseases"[All Fields] OR ("autoimmune"[All Fields] AND "disease"[All Fields]) OR "autoimmune disease"[All Fields])) OR ("free"[All Fields] AND ("triiodothyronin"[All Fields] OR "triiodothyronine"[MeSH Terms] OR "triiodothyronine"[All Fields] OR "triiodothyronines"[All Fields]) AND ("level"[All Fields] OR "levels"[All Fields])) OR ("free"[All Fields] AND "T3"[All Fields] AND ("level"[All Fields] OR "levels"[All Fields])) OR ("free"[All Fields] AND "tetraiodothyronine"[All Fields] AND ("level"[All Fields] OR "levels"[All Fields])) OR ("free"[All Fields] AND "T4"[All Fields] AND ("level"[All Fields] OR "levels"[All Fields])) OR (("thyroid hormones"[MeSH Terms] OR ("thyroid"[All Fields] AND "hormones"[All Fields]) OR "thyroid hormones"[All Fields] OR ("thyroid"[All Fields] AND "hormone"[All Fields]) OR "thyroid hormone"[All Fields]) AND ("level"[All Fields] OR "levels"[All Fields]))) AND ("anti n methyl d aspartate receptor encephalitis"[MeSH Terms] OR ("anti n methyl d aspartate"[All Fields] AND "receptor"[All Fields] AND "encephalitis"[All Fields]) OR "anti n methyl d aspartate receptor encephalitis"[All Fields] OR "anti n methyl d aspartate receptor encephalitis"[All Fields] OR ("anti n methyl d aspartate receptor encephalitis"[MeSH Terms] OR ("anti n methyl d aspartate"[All Fields] AND "receptor"[All Fields] AND "encephalitis"[All Fields]) OR "anti n methyl d aspartate receptor encephalitis"[All Fields] OR ("anti"[All Fields] AND "nmda"[All Fields] AND "receptor"[All Fields] AND "encephalitis"[All Fields]) OR "anti nmda receptor encephalitis"[All Fields]) OR ("autoimmune diseases of the nervous system"[MeSH Terms] OR ("autoimmune"[All Fields] AND "diseases"[All Fields] AND "nervous"[All Fields] AND "system"[All Fields]) OR "autoimmune diseases of the nervous system"[All Fields] OR ("autoimmune"[All Fields] AND "encephalitis"[All Fields]) OR "autoimmune encephalitis"[All Fields])) | 2152 |
| Google Scholar | (encephalitis or anti-N-methyl-D-aspartate receptor encephalitis or anti NMDA receptor encephalitis or autoimmune encephalitis) AND (thyroid function) | 1670 |
| Cochrane Library | (encephalitis or anti-N-methyl-D-aspartate receptor encephalitis or anti NMDA receptor encephalitis or autoimmune encephalitis) | 891 |
| Clinicaltrials.gov | (encephalitis or anti-N-methyl-D-aspartate receptor encephalitis or anti NMDA receptor encephalitis or autoimmune encephalitis) | 6 |
| Science Direct | (encephalitis or anti-N-methyl-D-aspartate receptor encephalitis or anti NMDA receptor encephalitis or autoimmune encephalitis) AND (thyroid function) | 371 |

**QA**

| STUDY ID | SELECTION ^a^ | | | | COMPARABILITY ^b^ | OUTCOME ^c^ | | | QUALITY ASSESSMENT BASED ON AHRQ ^d^ |
| --- | --- | --- | --- | --- | --- | --- | --- | --- | --- |
|  | Representativeness of the exposed cohort | Selection of non-exposed cohort | Ascertainment of exposure | Demonstration that outcome of interest was not present at the start of the study | Comparability of cohorts on basis of design or analysis controlled for confounders | Assessment of outcome | Adequacy of the duration of follow up | Adequacy of completeness of follow up |  |
| Ma et al. 2018 | * | * | * | - | ** | * | - | * | Good |
| Chen et al. 2021 | * | * | * | - | ** | * | * | * | Good |
| Ji et al. 2021 | * | * | * | * | ** | * | * | * | Good |
| Lin et al. 20222 | * | * | * | - | ** | * | * | * | Good |
| Wang et al. 2022 | * | * | * | - | - | * | * | * | Fair |
| Qiao et al. 2022 | * | * | * | - | ** | * | * | * | Good |

^a,b,c^ The higher the number of asterisk (*), the better quality of a given criterion. The maximum asterisks possible for each domain are selection = 3, comparability =2, outcome = 3. ^d^The Newcastle Ottawa Scale for Cohort studies converted to AHRQ standards (good, fair, poor). AHRQ: Agency for Healthcare Research and Quality.

**
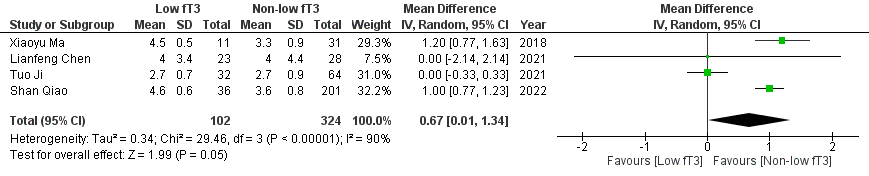
**

**Fig. S1. Forest plot depicting mean differences for mRS at admission before sensitivity analysis**

**
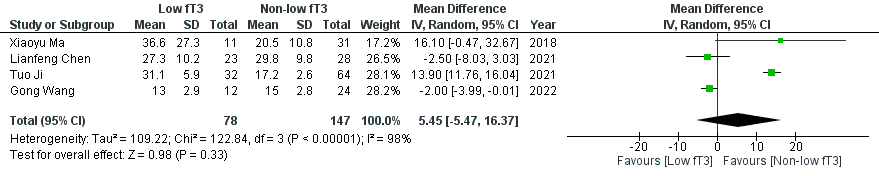
**

**Fig. S2. Forest plot depicting mean differences for length of stay at hospital before sensitivity analysis**

**
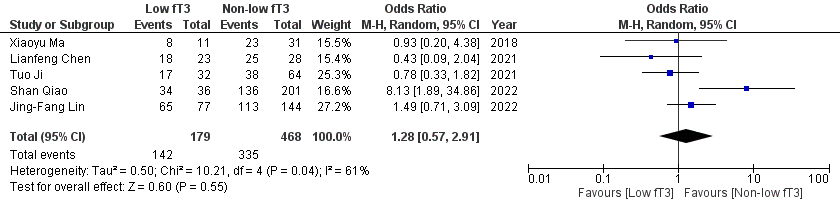
**

**Fig. S3. Forest plot depicting odds ratios for seizures before sensitivity analysis**

**
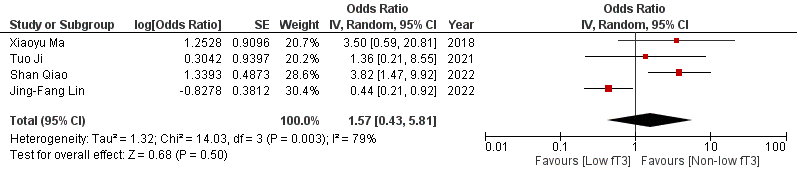
**

**Fig. S4. Forest plot depicting odds ratios for tumor presence before sensitivity analysis**

**
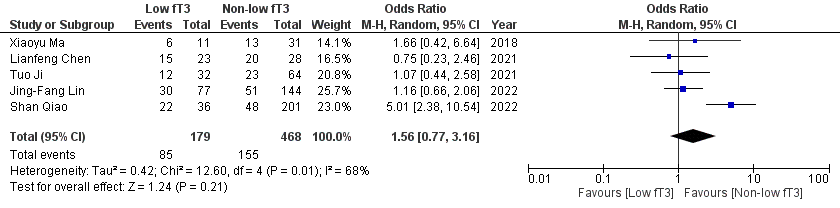
**

**Fig. S5. Forest plot depicting odds ratios for movement disorders before sensitivity analysis**

**
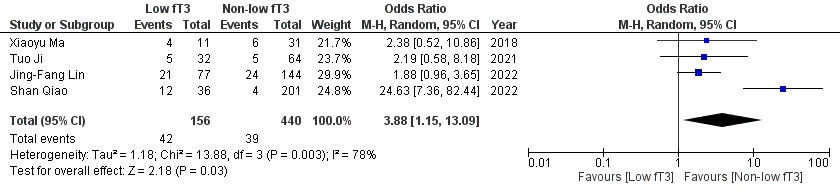
**

**Fig. S6. Forest plot depicting odds ratios for central hypoventilation before sensitivity analysis**
